# Integrated optical waveguide-based fluorescent immunosensor for fast and sensitive detection of microcystin-LR in lakes: Optimization and Analysis

**DOI:** 10.1038/s41598-017-03939-8

**Published:** 2017-06-16

**Authors:** Lanhua Liu, Xiaohong Zhou, James S. Wilkinson, Ping Hua, Baodong Song, Hanchang Shi

**Affiliations:** 1 Center for Sensor Technology of Environment and Health, State Key Joint Laboratory of ESPC, School of Environment, Tsinghua University, Beijing, 10084 China; 2 Optoelectronics Research Centre, Southampton University, Highfield, Southampton, SO17 1BJ UK

## Abstract

Nowadays, biosensor technologies which can detect various contaminants in water quickly and cost-effectively are in great demand. Herein, we report an integrated channel waveguide-based fluorescent immunosensor with the ability to detect a maximum of 32 contaminants rapidly and simultaneously. In particular, we use waveguide tapers to improve the efficiency of excitation and collection of fluorescent signals in the presence of fluorophore photobleaching in a solid surface bioassay. Under the optimized waveguide geometry, this is the first demonstration of using such a type of waveguide immunosensor for the detection of microcystin-LR (MC-LR) in lake water. The waveguide chip was activated by (3-Mercaptopropyl) trimethoxysilane/N-(4-maleimidobutyryloxy) succinimide (MTS/GMBS) for immobilization of BSA-MC-LR conjugate, which was confirmed to have uniform monolayer distribution by atomic force microscopy. All real lake samples, even those containing MC-LR in the sub-microgram per liter range (e.g. 0.5 μg/L), could be determined by the immunosensor with recovery rates between 84% and 108%, confirming its application potential in the measurement of MC-LR in real water samples.

## Introduction

Research activities on chemical and biochemical sensors have progressed dramatically over the past three decades. At present, much research work is focused on the development of systems capable of multi-analyte detection in a single sample, for environmental, clinical or security applications^1, 2^. Optical sensors have great potential in this field because of their ability to probe surface films using a range of optical phenomena while achieving low noise and high sensitivity. In addition, they have advantages in speed and permit *in-situ* sensing and real-time measurements. Optical sensors are also suitable for miniaturization and for remote and multi-analyte sensing. Another important feature of an optical sensor system is that it is substantially free from electromagnetic interference and has a reduced possibility of causing an explosion in a dangerous environment, compared to electrical transduction systems. Therefore, optical biosensors offer several advantages over laboratory-based systems when compared to other sensing systems.

Among these, waveguide-based evanescent wave fluorescent biosensors have attracted intensive attention because of their potential for easy miniaturization and their high sensitivity and selectivity^1, 2^. The evanescent wave provides the excitation energy to induce fluorophore emission which can then be detected and directly related to the analyte concentration in samples. In principle, the combination of evanescent wave excitation and fluorescent labeling offers both outstanding sensitivity and selectivity. The evanescent wave essentially confines the excitation power within a submicron distance from the sensor surface, providing the selectivity to excite only the fluorophores attached to the sensor surface, thereby minimizing the interference or contribution from the bulk phase^3^. Furthermore, the excitation light is waveguided away from the detection region, allowing simple discrimination of the fluorescence signal from the excitation light and achieving high sensitivities and low limits of detection (LODs)^3–6^.

Microcystin-LR (MC-LR) is one of the most toxic cyclic heptapeptide cyanotoxins released by cyanobacterial blooms in surface waters, for which sensitive and specific detection methods are necessary to carry out recognition and quantification^7^. Although several analytical techniques for microcystin detection such as ELISA, HPLC and LC-MS/MS etc. have already been established, the development of biosensors offers rapid and accurate detection, high reproducibility and portability^8^. Shi *et al*. reported an automated online waveguide-based evanescent wave fluorescent immunosensor for the detection of microcystin-LR, which adopted a rectangular glass chip with a polished 45° bevel on one endface for light coupling as the evanescent wave transducer^9^. Following this approach, a linear strip laser excitation beam was used to expand the multi-analyte analysis capability, allowing for simultaneous measurements of up to twenty-four analytes; subsequently, the target compound of MC-LR was selected as a paradigm to validate the sensitivity of the biosensor, achieving a LOD of 0.67 µg/L^10^. However, single mode waveguides are of great interest compared with the extremely multimode waveguides demonstrated in the above studies^9, 10^, because of several specific advantages^11^. For example, single mode planar waveguides (i) yield much higher surface intensity than multimode waveguides for a given waveguide power^12^, allowing high signal strength for low laser power, (ii) provide very stable and well-defined surface intensity distributions and unique optical velocity leading to much greater stability and hence low noise, and (iii) allow stable monolithic integration of multiple functions leading to multisensor integration and potentially on-chip processing. As far as we know, there are no other studies of using single mode waveguide-based fluorescent biosensors for MC-LR measurement. The indirect competitive assay is commonly adopted in immunoassays to create a stably regenerable biosensing chip surface, which is especially critical for the application of such an immunosensor for on-line and semicontinuous operation^9, 10, 13–18^. Extensive efforts have been devoted to control the configuration and orientation of functional molecules on the chip surface, forming a well-accepted conclusion that a monolayer of bioactive solid surface enhances the performance of immunosensors^16^. However, there is still a dearth of direct imaging data on the biosensitive surface to confirm monolayer formation.

Herein, we reported a 32-analyte integrated optical fluorescence-based multi-channel sensor, and its integration to an automated biosensing system. A beam-propagation model which simulates the propagation of light throughout the waveguide layout, including surface intensity distribution for fluorescence excitation, has been established allowing design optimization of waveguides for immunosensing applications. Moreover, we describe the first demonstration of the use of such a waveguide immunosensor for the detection of Microcystin-LR in lakes. An indirect competitive immunoassay is adopted with the MC-LR-protein conjugate immobilized on the chip surface, which is investigated using the Atomic Force Microscopy (AFM) technology.

## Results

### Waveguide Geometry: Simulation and Optimization

**Figure 1:**
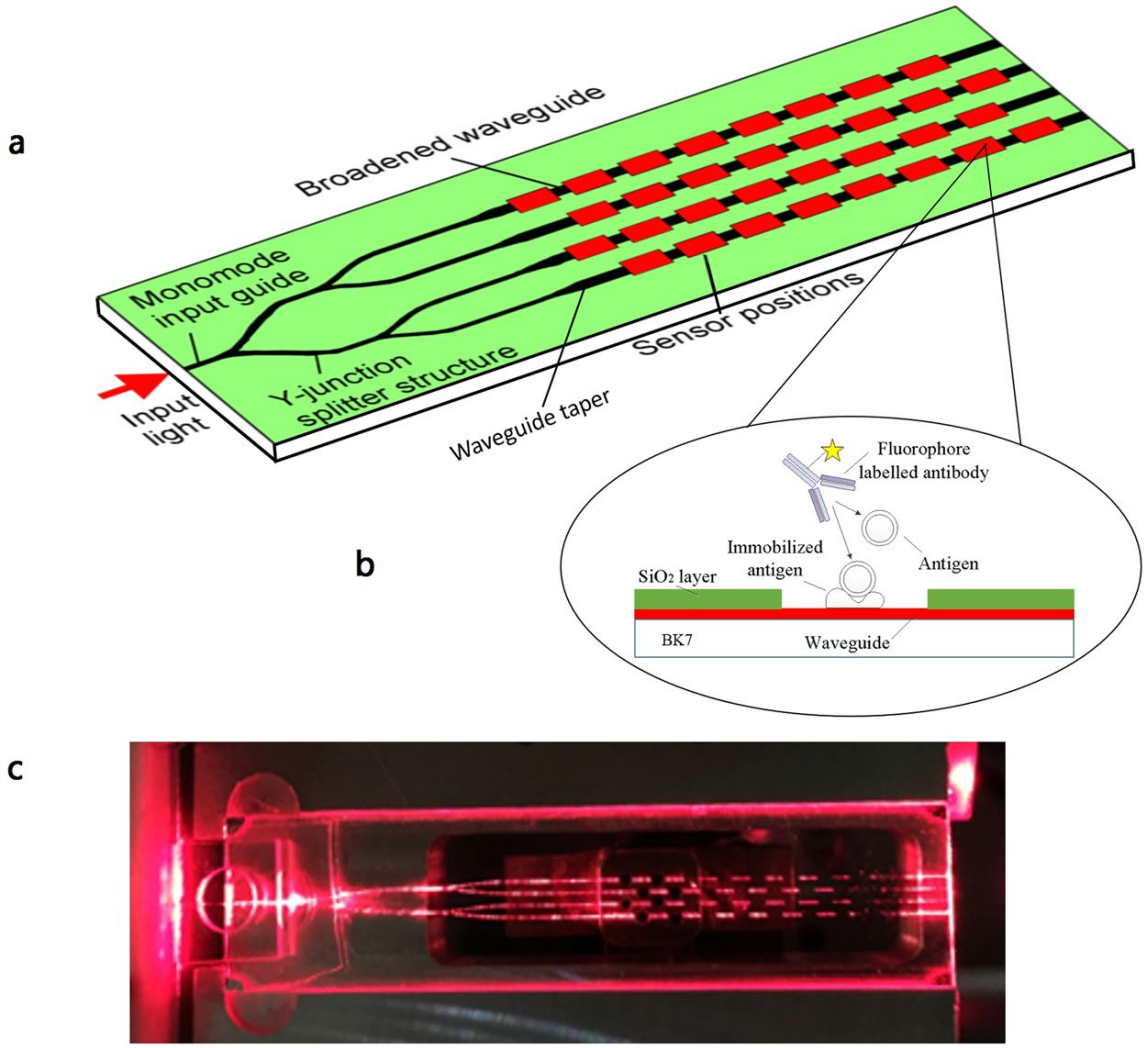
(**a**) Schematic diagram of the sensor layout; (**b**) A cross-sectional view along one of the sensing patches, showing the waveguide, isolation layer and location of the surface chemistry; (**c**) A photographic image of light propagation along the waveguide chip (Photo by Lanhua Liu).

Herein, a fibre-pigtailed waveguide chip consisting of a channel waveguide circuit which distributes excitation light to 32 separate sensing patches on the surface is presented, following the layout shown in Fig. 1a. In order to obtain low-loss, single-mode waveguides with a modal spot size similar to that of optical fibre at 635 nm, potassium ion-exchange in BK7 glass substrates was selected to give a good index match to optical fibre. A tapered waveguide section is introduced into the chip design to address the dilemma that the high surface excitation intensity will be accompanied by photobleaching of the dye molecules, which would reduce the sensitivity of the device, due to rapid decay of the signal^19^. In our design, an adiabatic taper section is employed to broaden the waveguides in order to reduce the power density of excitation radiation at the surface of the waveguide, while increasing the area over which the fluorescent-tagged molecules are exposed to the evanescent field. In this way, the peak emitted fluorescence power is maintained (as the product of area and surface power density is maintained) but the photobleaching rate is reduced, allowing longer acquisition time and hence improved signal to noise ratio and lower LOD.

A silica isolation layer of thickness 1 μm is used to coat the waveguide surface, with areas of 1500 μm × 300 μm opened as sensing windows. The intensity at the surface of the isolation layer is negligible compared with that at the waveguide surface within the window, effectively isolating the chip from the analyte outside these windows. A cross-sectional view along one of the sensing patches, showing the waveguide, isolation layer and location of the surface chemistry is shown in Fig. 1a and b photographic image of light propagation along the waveguide chip is shown in Fig. 1c.

#### Modal intensity profiles and fibre to waveguide coupling efficiency

The device we discuss here would ultimately be used in a portable instrument which requires the sensor chip to be pigtailed with a fibre, allowing easy connection and coupling of light from a laser source. The fibre-to-waveguide coupling efficiency was optimized as a first step in optimizing the sensor chip design.

Waveguide modal intensity profiles were measured using a CCD camera and compared with that of the fibre to be used for pigtailing, and the optimum fabrication conditions was selected to give a minimum coupling loss^20^; Subsequently, fibre-waveguide coupling efficiency measurements were made on these waveguides to confirm the optimum fabrication conditions for fibre coupling. As a result a 2.5 µm photolithographic mask opening was chosen for the waveguides in order to maximize the fibre/waveguide coupling efficiency at approximately 80%.

#### Waveguide surface intensity

Having established the single mode input waveguide design for efficient fibre coupling, the modal surface intensity in the sensing window regions must be optimised for efficient fluorescence excitation before designing the overall waveguide layout. The laser pump or excitation intensity at the waveguide surface directly affects the excitation efficiency of the fluorescent dye, thus affecting the fluorescent power and sensitivity of the sensor. The fluorescence emission power density, *I*_*emitted*_, is given by:

1 $${I}_{emitted}=D\times \eta \times ({\lambda }_{P}/{\lambda }_{e})\times {\sigma }_{a}\times {I}_{s}$$

In which *D* is the molecule surface density, *η* is the quantum efficiency of the fluorescent dye (for Cy5.5, *η* = 0.28), *λ*_*p*_ is the pump wavelength of 635 nm and *λ*_*e*_ is the emission wavelength of Cy5.5 at 700 nm, *σ*_*a*_ is the absorption cross-section of Cy5.5, which is 3.6 × 10^−20^ m^2^ and *I*_*s*_ is the surface intensity, which is calculated by numerical simulation. It has been found using aqueous dye solutions for preliminary characterization that a detection limit of 10^−8^ M Cy5.5 solution results in sufficient detection limit for subsequent immunoassay^10^. The 10^−8^ M fluorophore solution will bring the same number of fluorophores into the excited volume as a dye molecule surface density of D ≈ 1.8 × 10^12^ (Cy5.5 molecules)·m^−2^.

**Figure 2:**
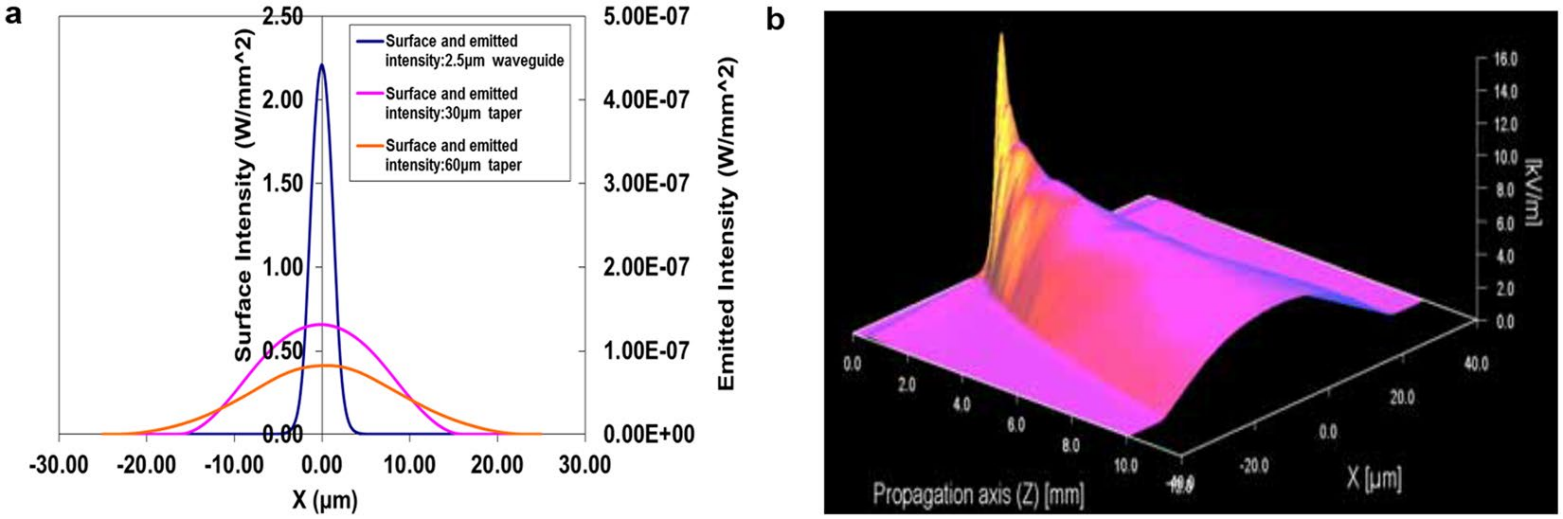
(**a**) Surface intensity of the start (2.5 µm) and the end of 30 µm and 60 µm tapered waveguides in the lateral direction and emitted fluorescence intensity; (**b**) Electric field strength at the surface of a waveguide tapered from 2.5 µm to 60 µm over 10 mm.

A beam propagation model was established to design adiabatic tapers to connect the single mode input waveguides to the sensing patches and to determine waveguide surface intensity distributions. The 3D beam propagation method (OlympIOs BPM) was used with the refractive index profile for potassium ion-exchange in BK7 glass. The taper used was parabolic in width without tapering of the depth and the length was set at 10 mm due to chip-size constraints. Waveguides tapering from 2.5 microns width at the input to 60 microns, 30 microns and 2.5 microns (untapered) widths at the output were modelled. Wider tapering was found to lead to significant excitation of higher-order modes in the wide sections, which would result in undesirable surface intensity fluctuations. Figure 2a shows the simulation result for waveguide surface intensity and estimated emitted fluorescence intensity for the untapered waveguides (2.5 µm), and at the ends of the 30 µm and 60 µm taper waveguides. It can be seen that as the waveguide width increases, the surface intensity and fluorescence power density decrease but cover a larger surface, as discussed above. To estimate the emitted intensity, a surface coverage of 18 × 10^12^ Cy5.5 molecules per m^2^, corresponding to a 10^−7^ M solution, and 1 mW input power into a single waveguide are assumed. Given the expected laser/waveguide coupling efficiency and the 1 × 4 splitter, this corresponds to approximately 5 mW laser power from the fibre. To illustrate the near-adiabatic tapering, Fig. 2b shows a surface plot of the electric field along the length of the 2.5 µm to 60 µm taper, where it is clearly shown that the mode remains in the the fundamental mode of the broadened waveguide.

The emitted fluorescent power can be calculated from Eq. 1 using the laser surface intensity in Fig. 2a by integration over the excited area, resulting in ~0.45 × 10^−9^ W fluorescent power in the case of the 30 µm wide waveguide over a 1.5 mm length, for a 10^−8^ M dye solution with 1 mW laser power in the waveguide.

We have estimated the maximum fractional pump power absorbed by dye molecules bound at a patch so that we can quantify the effect of binding on one patch upstream of another. Using the maximum surface dye density and the absorption cross-section and knowing the surface intensity normalized to the input power, this was estimated to be <0.1%, so that a binding reaction on one patch will have an insignificant effect on the excitation power at downstream sensing patches.

### Instrumentation

**Figure 3:**
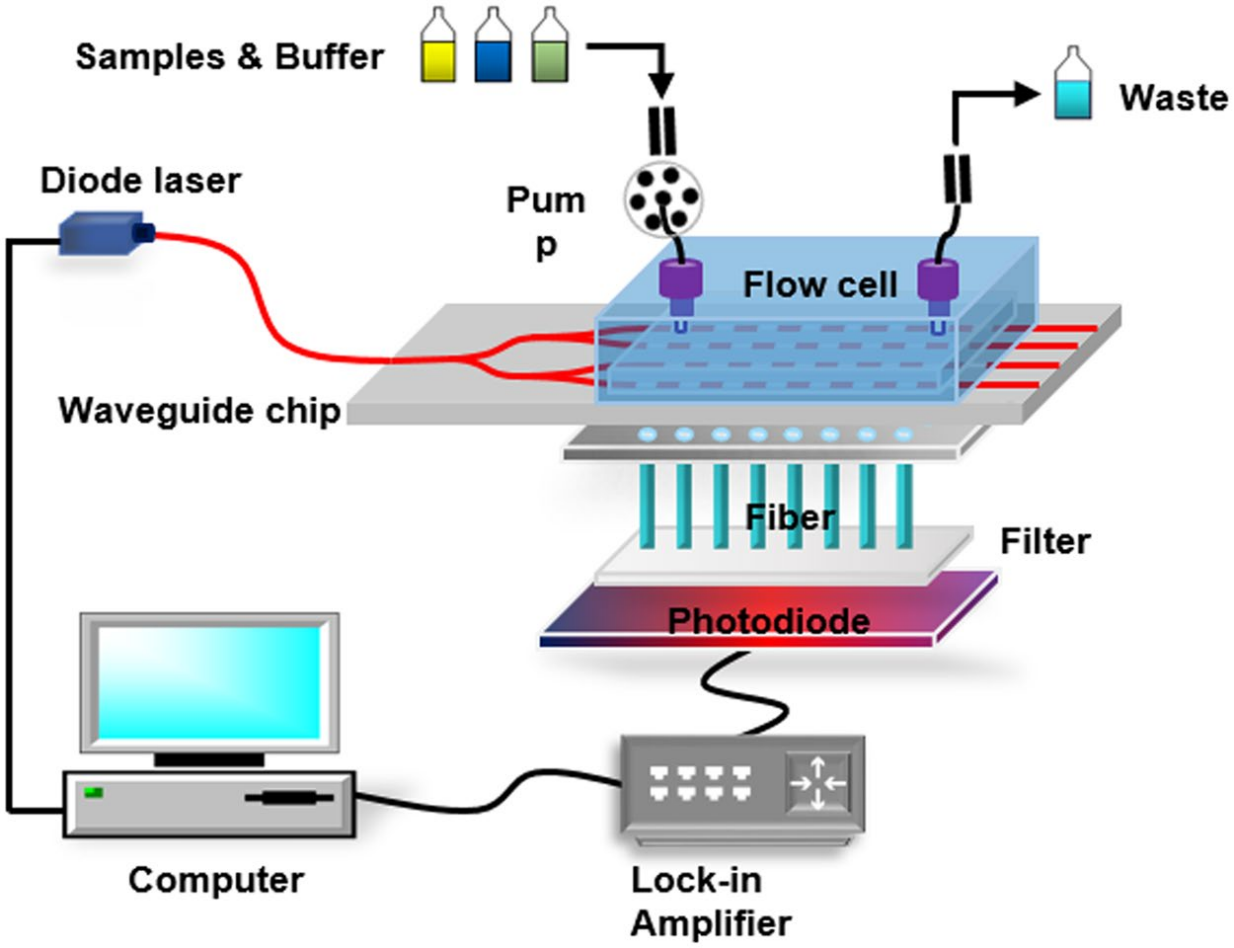
Schematic diagram of the integrated optical fluorescence multi-channel biosensor.

Figure 3 illustrates the experimental configuration of the multi-channel waveguide-based fluorescent evanescent wave biosensing platform. Briefly, light from a semiconductor laser emitting approximately 5 mW at 635 nm is coupled to the fibre-pigtailed multi-channel ion-exchange waveguide. The emitted fluorescent light is collected by 32 polymer fibres (NA = 0.46, 1 mm in diameter) located beneath the waveguide opposite to the biosensing surface (please see Supplementary Materials: design of the fibre collection system, Page 2). The end faces of the fibres are parallel to the chip surface. The fluorescent light is subsequently filtered by a high-pass filter to reject the scattered laser light. The fluorescent power is further processed and detected by photodiodes with Noise Equivalent Power (NEP) of 0.15 pW/Hz^−1/2^, through a lock-in amplifier, and the peak fluorescent power received is used as the characteristic signal associated with the concentration. The received power is related to the emitted fluorescent power by the collection efficiency, which takes into account the collection fibre area and numerical aperture and other geometrical factors, and is estimated to be 0.08% for the 30 µm waveguide^20^. If a minimum emitted power of 0.45 nW must be detected, this would require a minimum detected power on the photodiode of 0.36 pW, which is achievable with this detection system using a 1 Hz receiver bandwidth. A syringe pump, a six-way injection valve, a preincubation loop (1 mL), a solenoid valve, and a flow cell comprise the flow injection system. The antibody and the preincubation loop are stored in two individual thermostats, where the temperatures are maintained at 4 °C and 37 °C, respectively, to ensure the activity and stability of biological reagents. Fluid handling and data acquisition is fully automated and controlled by an embedded computer.

### Chip validation

**Figure 4:**
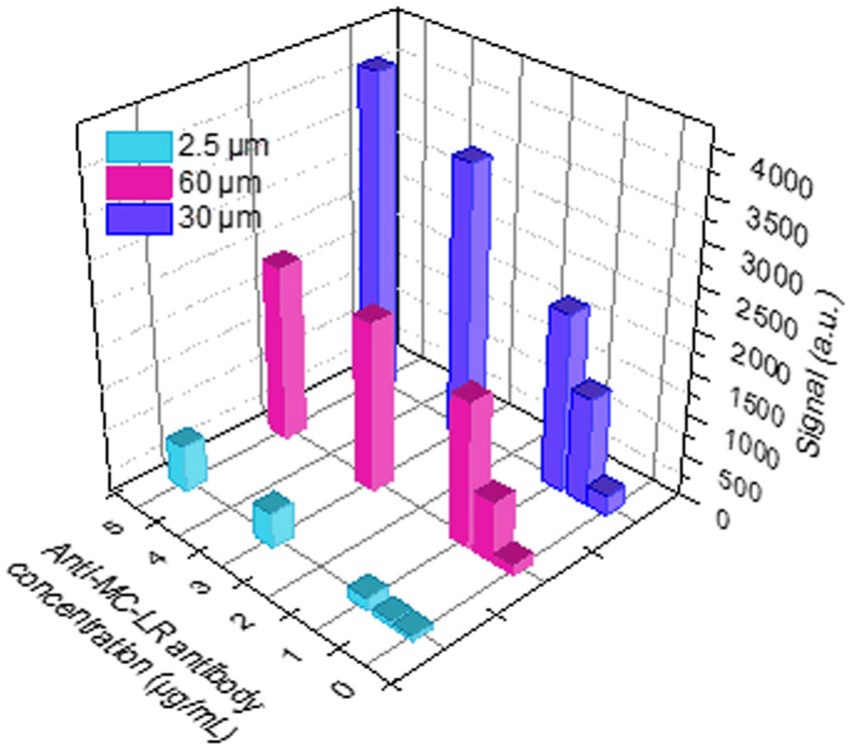
Fluorescence signals from untapered, 30 µm and 60 µm tapered waveguides, respectively, towards Cy5.5-labelled anti-MC-LR antibody solution at different concentrations.

In order to achieve the highest sensitivity for the device, we investigated three different waveguide widths for the immune chip with different concentrations of Cy5.5 labeled MC-LR antibody to determine response signals. From Fig. 4, it can be seen that when the width of the waveguide channel is 2.5 μm along its full length, the collected signal is very weak due to the strong photobleaching. With the 30 μm waveguides, the cross section of the waveguide is enlarged and the optical density is decreased. After the antibody is attached to the sensing region, the signal is relatively high. However, with 60 μm taper waveguide, the signal collected is lower due to reduced efficiency of collection into the fibre. Therefore, the waveguide chip with the 30 μm taper was selected for the subsequent tests. Based on the sensor system, Cy5.5 fluorescent dyes with different concentrations were tested at 32 detection sites of the waveguide chip. It was determined that 80% of the detection sites reached a detection limit for aqueous solution of fluorescent dye of 2.8 × 10^−9^ M, indicating that the sensitivity of the system is sufficient for the immunoassay. Figure S3 is the result of the response of 32 sensor sites with 30 µm waveguides to 10^−6^ M Cy5.5 solutions captured by the 32 fibres which indicates the system has good parallel operation and can achieve detection of up to 32 substances simultaneously.

### Immunoassay

**Figure 5:**
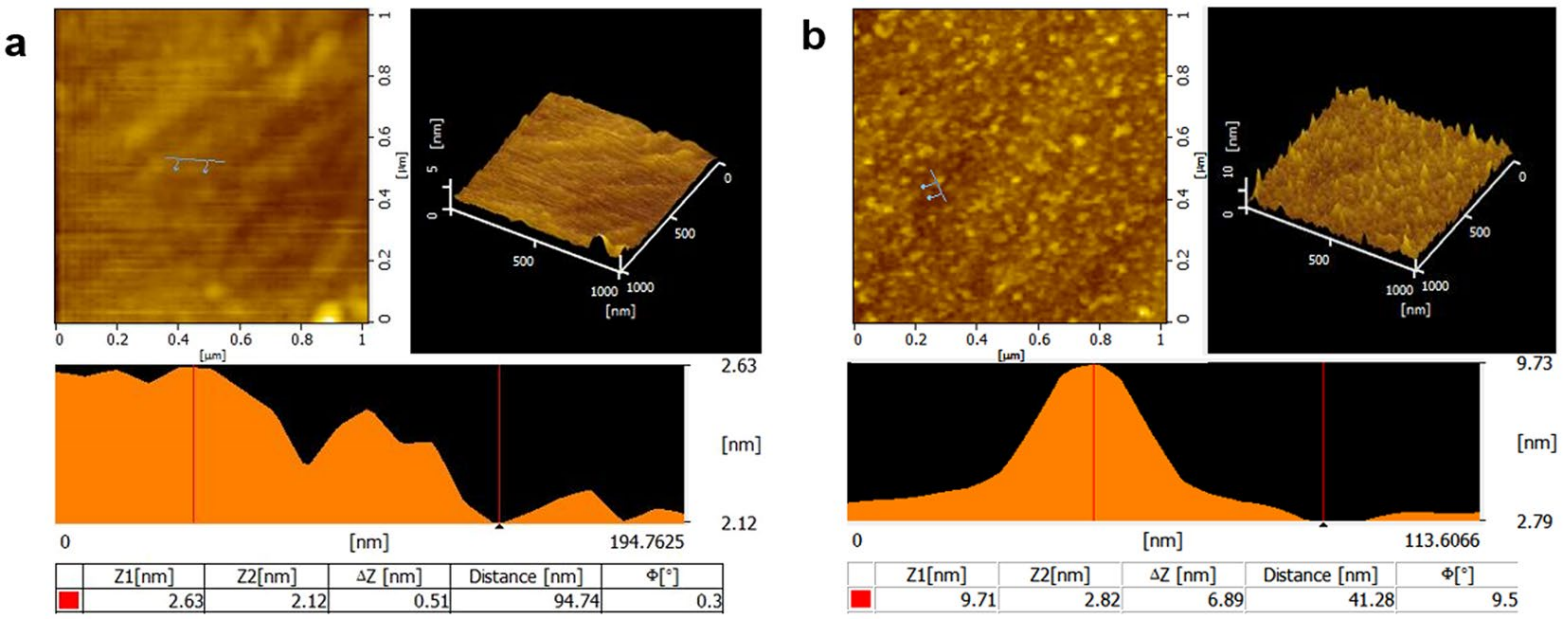
AFM topography images of (**a**) bare and (**b**) BSA-MC-LR conjugate modified waveguide chip, including a 1 µm × 1 µm plane AFM image (Higher left), a 3-D AFM topography image (Higher right), and cross-section height variations taken at a rough area (Lower: position indicated by blue line in the topographic images).

An indirect competitive immunoassay is adopted to enable regeneration of the transducer for reuse without loss of activity, thereby allowing semicontinuous water monitoring. A monolayer of BSA-MC-LR conjugate with good binding affinity and excellent long-term stability will form on the chip surface, thereby providing more effective binding of fluorophore-labeled antibody compared with non-monolayer-based immunosensors. AFM images of the bare and chemically modified waveguide chip surfaces are presented in Fig. 5. The surface roughness of the waveguide chip increased significantly as a result of the covalently coated BSA-MC-LR conjugate. Extensive analysis of the AFM topography cross-sections shows that the bare waveguide displayed the height variation of 0.51 nm; however, the value increased to 6.89 nm after chemical modification. Considering that the diameter one BSA molecular was about 7.2 ± 0.2 nm^21^, the AFM images clearly revealed that a monolayer of BSA-MC-LR conjugate successfully formed on the chip surface.

**Figure 6:**
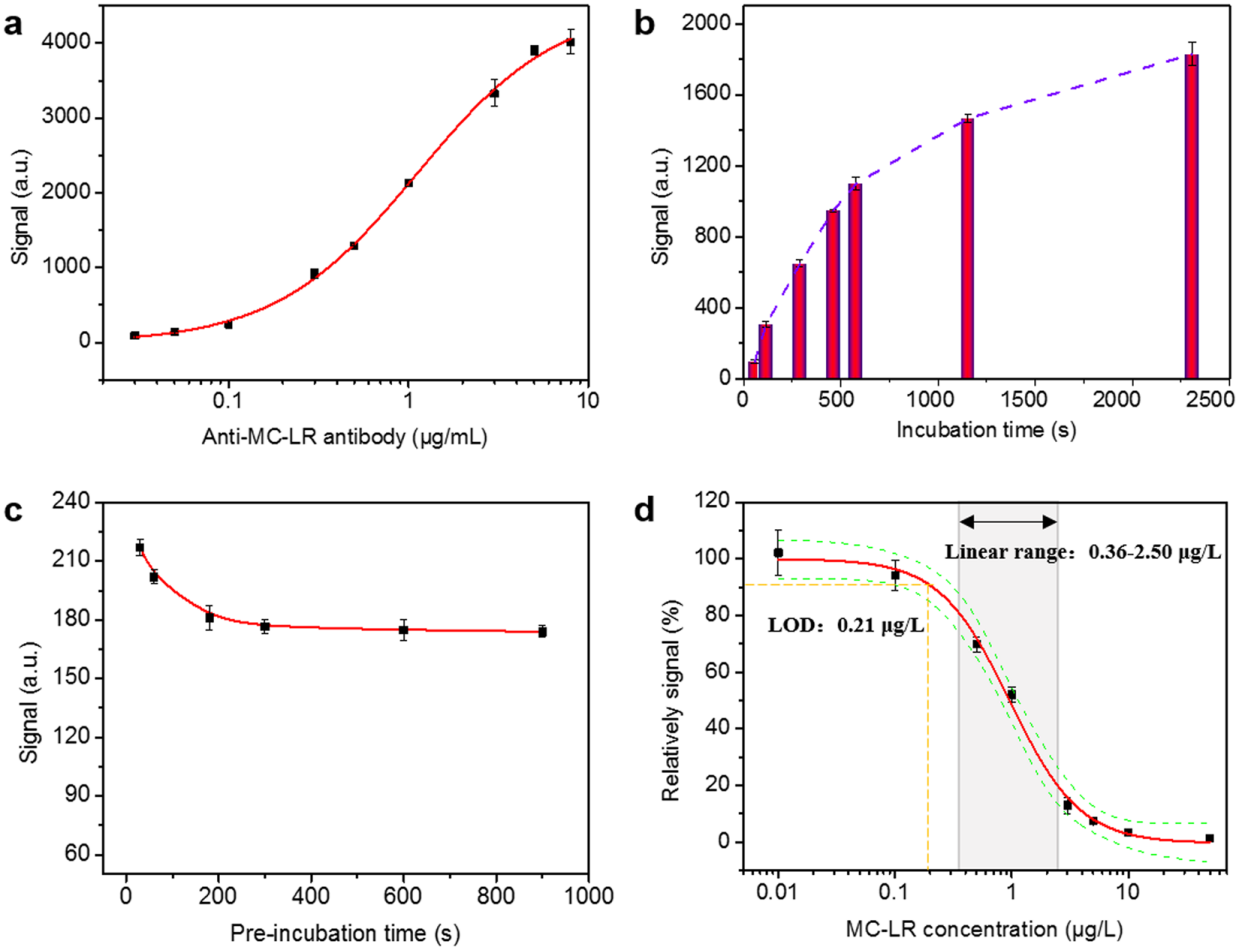
Relationships between (**a**) the Cy5.5-labeled MC-LR antibody concentration, (**b**) the incubation time, (**c**) the preincubation time and fluorescence signals, respectively; (**d**) Typical calibration curve in triple measurements (Green line represents 95% confidence range) for MC-LR by using the immunosensor.

In established biosensors for indirect competitive immunoassay, several factors are critical for device performance, including the preincubation time of labeled antibody and free antigen in samples, the incubation time when the preincubated mixture comes into contact with the biosensor surface, and the concentrations of immobilized antigen and labeled antibody. Among these factors, the concentration of labeled antibody is an important factor in immunoassays because it strongly affects the LODs and working ranges of immunosensors^13, 22, 23^. Figure 6a shows the relationship between the Cy5.5-labeled MC-LR antibody concentration and collected fluorescence signals. Through curve-fitting with the logistic function embedded in Origin Software, the linear range between the labeled antibody concentrations and the fluorescence signals was 0.31–3.9 μg/mL. The optimized labeled antibody concentration was selected to be 0.3 μg/mL anti-MC-LR antibody, close to the minimum value of the linear range for achieving both of the high sensitivity and low cost. Figure 6b shows the relationship between the incubation time and collected fluorescence signals. Not surprisingly, the signals increased with the increased incubation time, however, the rate of increase slowed significantly after 600 s. As the nonequilibrium state for the surface-based immunoassay may be used for measurement to shorten the detection time^9^, the optimum incubation time was selected to be 600 s. The fluorescence signals reached a plateau when the preincubation time was more than 300 s as shown in Fig. 6c, indicating that the immunoassay between the antibody and antigen in samples had reached equilibrium. Therefore, 300 s was used as the incubation time for this biosensor. Under the optimized detection conditions, the entire test cycle time is shown in Figure S4. The peak value with the laser on less the average value during the baseline acquisition is used as the immunosensor signal for subsequent analysis, such as in Figs 4 and 6. Figure 6d shows the typical calibration curve for this immunosensor towards MC-LR in series concentrations. The linear dynamic response range was 0.36 μg/L–2.50 μg/L with a detection limit (LOD) of 0.21 μg/L. Moreover, a complete test cycle of the immunosensor described herein is obtained within 20 min.

### Regeneration and reusability

Creating a regenerated biochip surface is critical for cost-effective, on-line and semi-continuous water monitoring. In the indirect competitive immunoassay described herein, the BSA-MC-LR conjugate, which is tolerant of a harsh regeneration environment^13, 24^, is covalently coated on the chip surface. Figure S5 illustrates the signal recovery after 20 consecutive determinations when the regeneration solution of 0.5% SDS (pH = 1.9) was used at a constant flow rate of 1 mL/min. With the use of the regeneration agent, the surface regeneration was conducted up to 100 times with less than 10% decrease in the signal for lake water detection, indicating no significant degradation of the surface chemistry.

### Recovery study

A recovery study was performed, in triplicate, using two real lake water samples taken from Fuhai lake and Beihai lake in Beijing, respectively, spiked with three different standard concentrations (0, 0.5 and 1 µg/L) of MC-LR. Before the measurement, the real lake water samples were filtered through a 0.45 μm filter. The concentrations measured were compared with the concentrations added and the results are summarized in Table 1. This shows that the average recoveries vary from 84% ± 7% to 108% ± 6%, demonstrating the satisfactory accuracy of the biosensor and confirming the application potential of our method to measure MC-LR in real lake samples.

**Table 1:** Recovery of MC-LR in real lake samples using the immunosensor (n = 3).

Sample	Spiked MC-LR (µg/L)	Detected MC-LR (µg/L)	Recovery %	Coefficient variation (CV) %
Fuhai lake	0	/	/	/
	0.5	0.42 ± 0.03	84	7
	1	0.98 ± 0.09	98	9
Beihai lake	0	/	/	/
	0.5	0.45 ± 0.05	90	9
	1	1.08 ± 0.06	108	6

“/”means not detectable.

## Discussion

New trends in water environmental monitoring highlight the need to develop tools for rapid, low-cost, routine and on-line contaminant detection for protecting the safety of water sources, risk identification and early warning for accidental water pollution. Based on its performance compared with laboratory-based technologies, the biosensor platform emerges as a suitable tool to detect large classes of compounds found in water environments^25^.

Herein, we have reported a 32-analyte optical fluorescence-based multi-channel waveguide biosensor integrated with fluidics and signal control and processing. An extensive study was undertaken theoretically and experimentally in order to optimize the sensor chip design, fabrication and sensing system. A taper width of 30 µm was found to be optimum and was chosen for the final waveguide design. The surface intensity in the sensor region was also studied in depth, based upon beam propagation method simulation of waveguides which had been optimized for fibre to waveguide coupling efficiency. A low loss, high signal strength and robust optical transducer for multiple parallel fluorescence immunoassay was realized.

The surface immunochemistry used in this research was based on an indirect competitive immunoassay that requires the analyte derivatives covalently bound to the transducer surface, thereby bringing highly stable regeneration capacity. The AFM images of a waveguide chip before and after covalently chemical modification revealed that a monolayer of BSA-MC-LR conjugate successfully formed on the chip surface. This allows for more than 100 regeneration cycles, consistent with the previous results reported by other researchers^10, 15^. Under optimized detection conditions, single and multiplexed detection of contaminants in water samples are expected to be realized.

The target compound of MC-LR was chosen as a paradigm to validate the sensitivity of the biosensor because accidental animal poisoning and human diseases, even death, due to exposure to MCs by way of drinking and surface water have been much reported^26, 27^. In this regard, the World Health Organization (WHO) has proposed an MC-LR guideline maximum value of 1 µg/L in drinking water in order to minimize public exposure to MCs^28^. In 2002, China introduced the guideline value for MC-LR in drinking water with a recommended limit of 1 μg/L. Therefore, there is a great need to establish the cost-effective, reliable and sensitive methods for the detection of MC-LR within natural systems for protecting environment and public health. By using the home-made anti-MC-LR monoclonal antibody (MC-LR-MAb, 8C10), the linear dynamic response range was 0.36 μg/L-2.50 μg/L with a LOD of 0.21 μg/L. This is sufficiently sensitive for detection of MC-LR at the maximum concentration levels as established by the Chinese government, WHO and other countries. The entire test cycle time is no more than 20 min. All real lake samples, even those containing MC-LR in the sub-microgram per liter range could be determined using the biosensor with recovery rates between 84 and 108%. If there are sufficient available monoclonal antibodies with good performance, such as high sensitivity, selectivity and negligible cross-reactivity, the multi-analyte detection can be realized on one chip via immobilizing the different antigen-protein conjugates on the separate sensing patches. Moreover, the biosensor can perform simultaneous testing of multiple water samples for one analyte through the design of independent flow cells along with flow injection system.

Overall, the biosensor reported herein can serve as a common platform for the immunoassay of environmental contaminants, providing a reliable, feasible and cost-effective alternative to laboratory-based analytical technologies.

## Methods

### Materials and reagents

The fluorescent dye Cy5.5 N-hydroxysuccinimide (NHS) ester were purchased from GE Healthcare Life Sciences. Labeling of the anti-MC-LR-antibody with dye Cy5.5 was performed based on the method as previously described by Mujumdar *et al*.^29^. The hapten conjugates were synthesized based on the procedure previously reported^17^. The Cy5.5-labeled antibody and the hapten conjugates were purified and stored at −20 °C in small aliquots for use. All chemicals were analytical grade and were used without further purification. Deionized water was used throughout the experiments. 1 mg/L MC-LR stock solutions were prepared in methanol solvent and stored at −20 °C before use. Phosphate buffered saline (10 mM PBS, pH 7.4) was prepared using deionized water (18.2 Ω · cm). All the stock solutions were diluted to a series of concentration levels using the 10 mM PBS buffer solution.

### Waveguide chip fabrication by potassium ion-exchange

Multi-channel sensor chips were fabricated by potassium ion-exchange in BK7 glass, since BK7 is of good optical quality and exhibits low fluorescence, and the process produces low loss waveguides. The overall chip dimensions were 67 mm × 15 mm, the length allowing low-loss bends and tapers and the width dictated by ease of handling. On each chip the monomode input waveguide was split into four monomode waveguides to provide equal power division and parabolic taper waveguides were introduced into each waveguide branch after the Y-junction splitters in order to reduce the optical power density at the waveguide surface, and hence to reduce the rate of fluorophore photobleaching. Separate chips were made with no tapering, with tapering to 30 µm and with tapering to 60 µm.

The waveguide circuit was defined by opening tracks in an aluminium film deposited on the glass substrate, ranging from 2.5 µm wide for a single mode waveguide at 635 nm to 60 µm wide for the tapers, using conventional photolithography.

Ion exchange was carried out by immersing the masked substrates in KNO_3_ at 400 ± 5 °C for 2 h to produce single mode channel waveguides with good coupling to optical fibre.

A silica layer thickness of 1 µm was deposited on top of the waveguides by RF sputtering and the sensing windows were defined by lift-off photolithography. The intensity at the surface of the isolation layer is negligible, isolating the chip from the analyte outside the window regions.

The ends of the chips were polished to allow fibre coupling. A fibre pigtail was permanently bonded to the input end of the sensor chip with UV-curing epoxy.

### Immunoassay and evaluation

An indirect competitive immunoassay for the trace concentration of MC-LR detection is developed and stepped as follows. The MC-LR-BSA conjugate is covalently immobilized on the chip surface by a similar procedure described by Long *et al*.^17^ (Figs 1b and S6). When performing the test cycle, 0.8 mL of sample solution and 0.2 mL of 0.3 μg/mL Cy5.5-labelled antibody solution (in 10 mM PBS containing 5.0 mg/mL BSA and 0.1 mg/mL thiomersal) is firstly transferred to the preincubation loop for 5 min to make the antibody-binding sites occupied with the analyte. Subsequently, the mixture is delivered into the flow cell. Antibodies with free binding sites remaining interact with the coated antigen immobilized on the biochip for 10 min. To reduce the effect of free antibody in solution and its non-specific adsorption on the detection result, the fluorescence signal is detected after the mixture is washed with PBS solution. The amount of antibody coupled on the chip surface is inversely correlated to the concentrations of MC-LR in samples, which can be reflected by the fluorescence signal.

The signal intensities were fitted to a four parameter logistic equation^10^,

2 $${\rm{A}}=\frac{{{\rm{A}}}_{1}-{{\rm{A}}}_{2}}{1+{(x/{x}_{0})}^{p}}+{{\rm{A}}}_{2}$$

where A is fluorescence intensity, *x* is the MC-LR concentration; A_1_ is the upper asymptote and A_2_ is the lower asymptote (background signal) to the titration curve; *x*_0_ is the analyte concentration at inflection and *p* is the slope at the inflection point. The quantitative detection range is defined as the signals from 20% to 80% of the signal difference region (A_1_ − A_2_), which is defined as a linear range. The limit of detection (LOD) is determined using the 90% of the signal difference region (A_1_ − A_2_)^17^.

## Electronic supplementary material

Supplementary Materials
