# Supplementary material for: Integrated optical waveguide-based fluorescent immunosensor for fast and sensitive detection of microcystin-LR in lakes: Optimization and Analysis
Source: Sci Rep. 2017 Jun 16;7:3655. doi: 10.1038/s41598-017-03939-8 (PMC5473886; doi:10.1038/s41598-017-03939-8)
Supplement: Supplementary file 1 — Supplementary Materials [file 41598_2017_3939_MOESM1_ESM.pdf]

Supplementary Materials:

Integrated optical waveguide-based fluorescent immunosensor for fast and sensitive detection of microcystin-LR in lakes: Optimization and Analysis

Lanhua Liu[a], Xiaohong Zhou[a*], James S. Wilkinson[b], Ping Hua[b**], Baodong Song[a], Hanchang Shi[a]

a.Center for Sensor Technology of Environment and Health State, Key Joint Laboratory of ESPC, School of Environment, Tsinghua University, Beijing 10084, China

b.Optoelectronics Research Centre, Southampton University, Highfield, Southampton, SO17 1BJ, UK

[*]xhzhou@mail.tsinghua.edu.cn;[**]ph2@orc.soton.ac.uk

Design of the fibre collection system

We design a coppery base according to the relative positions of sensing points (as shown in Figure S1). The 3D design of coppery base can be found in Figure S2. The plastic fibers will be inserted into the holes of base for fluorescence collection. The base is then connected to a flat plate and controlled by a three-dimensional manipulator. The relative position between the base and waveguide can be adjusted by the manipulator or that controlling the waveguide.

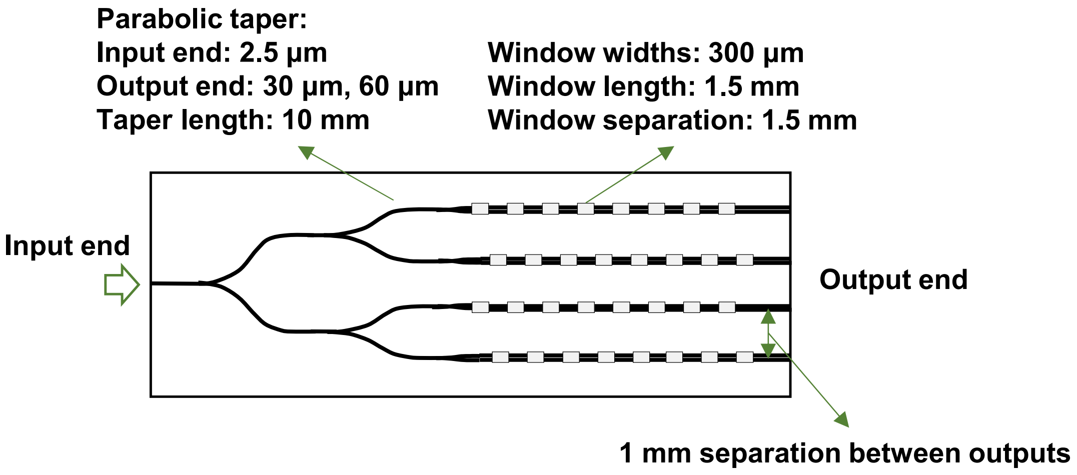

Figure S1 Layout of integrated fluorescent waveguide

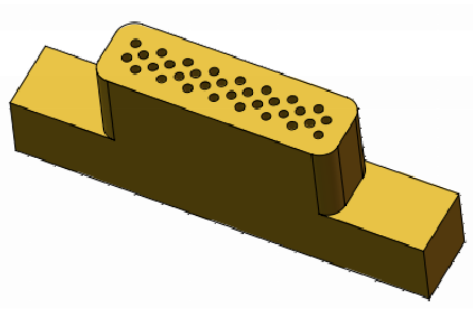

Figure S2 3D structure of coppery base for insertion of plastic fibres

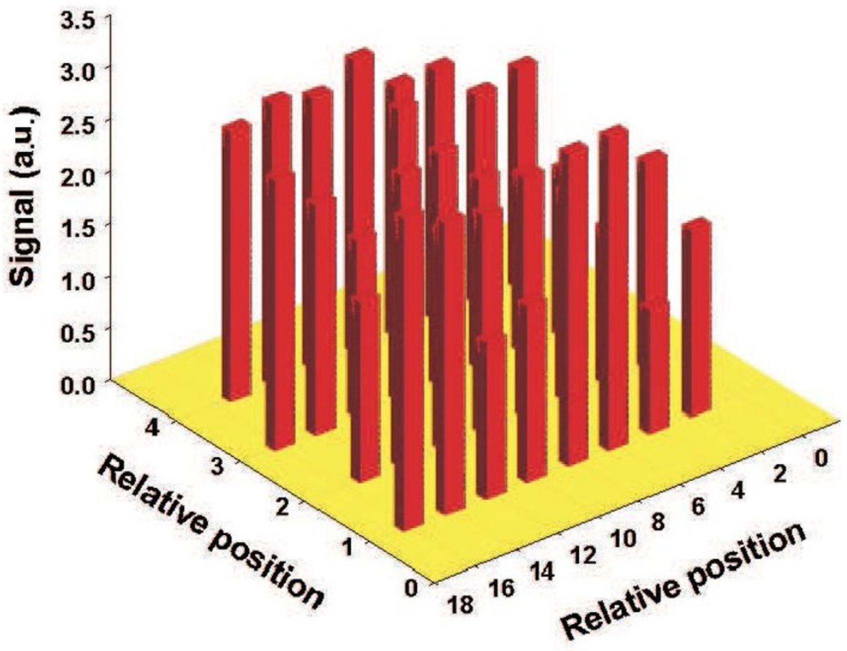

Figure S3 Mapping of the uniformity of the 32 patches with 10^{-6} M Cy5.5 dye on the sensor chip

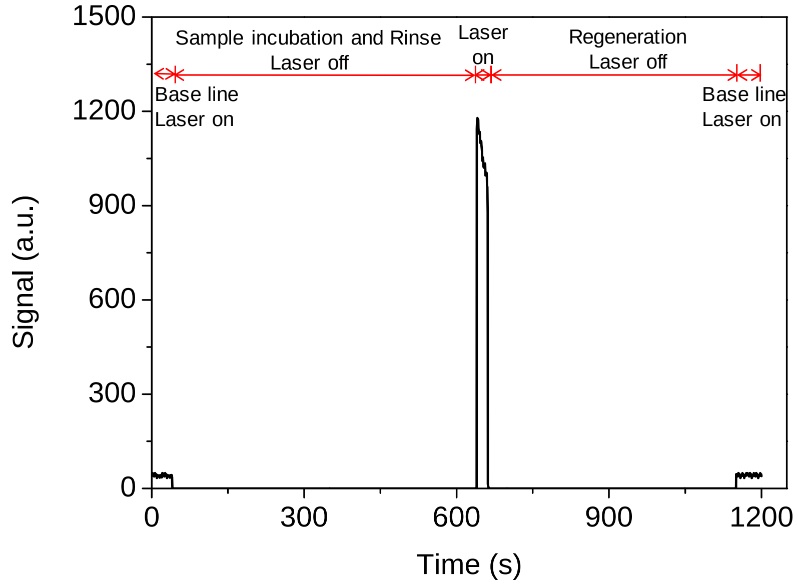

Figure S4 Entire test cycle of the immunosensor for MC-LR detection with signals collected when the laser is on

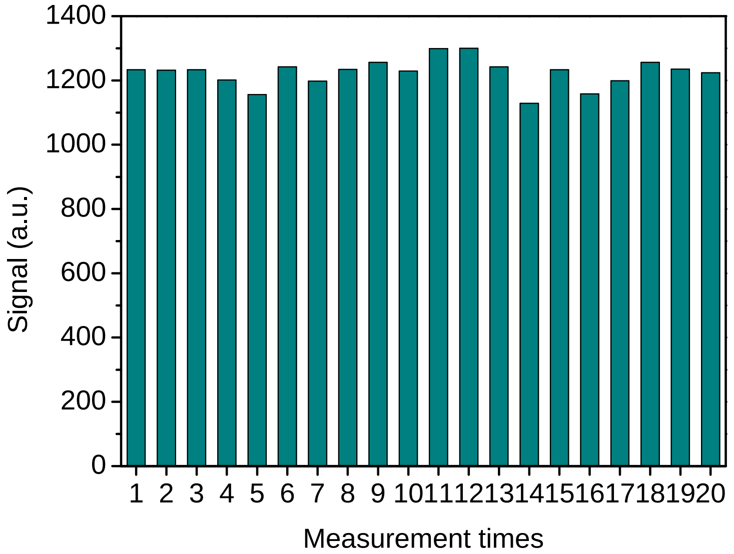

Figure S5 Signal recovery after 20 times of measurements regenerated with 0.5% SDS solution at pH = 1.9

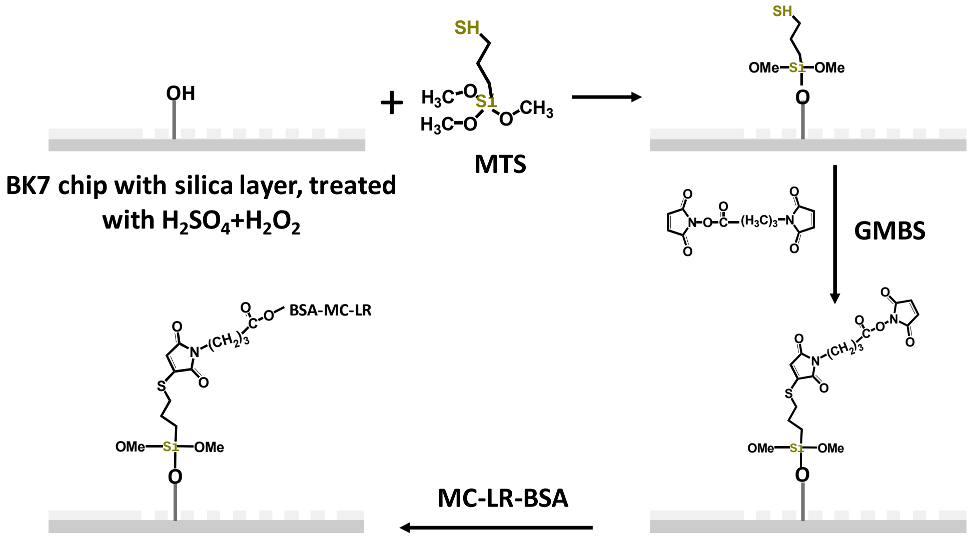

Figure S6 Immobilization schematic of hapten conjugate MC-LR-BSA on the chip surface

Briefly speaking, the hydroxyl groups were firstly introduced on the chip surface by exposed in piranha reagent (75% sulfuric acid and 25% hydrogen peroxide) for over 30 min. After washing with deionized water and dried in N_2, the chip was treated with 2% (v/v) of MTS in toluene for 2 h at room temperature (RT) to form a reactive silane layer. After removing the excess MTS with toluene, the thiol group of silane was then allowed to react with the maleimide function of the bifunctional crosslinker GMBS (2 mg/mL in ethanol) for 1 h at RT. Then, the ester moiety of the GMBS allowed the covalent linking of the MC-LR-BSA protein conjugate on the specific binding sites via the amino groups for overnight at 4 ℃. Finally, the chip was immersed in the BSA solution (2 mg/mL) for 1 h to block its non-specific binding sites.
